# Y-haplotypes and idiopathic male infertility in an Indian population

**DOI:** 10.4103/0971-6866.50865

**Published:** 2009

**Authors:** Kiran Singh, Rajiva Raman

**Affiliations:** Department of Molecular and Human Genetics, Banaras Hindu University, Varanasi, India; 1 Cytogenetics Laboratory, Department of Zoology, Banaras Hindu University, Varanasi, India

**Keywords:** Male infertility, single nucleotide polymorphism, Y-haplotypes

## Abstract

Infertility being a multifactorial disorder, both genetic and environmental factors contribute to the etiology of infertile phenotype. Chromosomal anomalies and Y-microdeletion are the established genetic risk factors of male infertility. Y-haplotypes has been found as risk factor for male infertility in certain populations, though in certain others no association has been reported, suggesting a population-specific association of these variations with male infertility. In a case-control study, 165 azoo-/oligospermic patients and 200 controls were haplotyped for certain Y-haplogroups for a possible association with idiopathic male infertility in an Indian population. Analysed Y-haplogroups showed no association with infertile phenotype. Thus this genetic factor is not a risk for infertility in the studied Indian population but that does not rule out the possibility of any of them, to be a risk in other populations.

## Introduction

The human Y chromosome contains over 60 million nucleotides, and act as a genetic determinant of the male characteristic features. Though rearrangement or sequence variation in these genes is expected to lead to impaired spermatogenesis and reduced sperm count,[1,2] seldom has a mutation in a single gene been associated with nonobstructive male infertility. On the other hand, the genetic cause of idiopathic azoospermia and oligospermia is predominantly associated with chromosomal aneuploidy, rearrangements, and microdeletions in the AZF region of the Y-chromosome.[3,4] The lack of inter-chromosomal recombination in larger part of the Y-chromosome facilitates accumulation of a variety of slow mutating binary markers such as SNPs. Variation in their relative frequency in different populations gives rise to diverse chromosomal haplotypes.[5] A few studies have shown an association of certain Y-haplogroups with low sperm count and azoospermia in certain populations which fortifies the idea that selection processes are still active on the Y-chromosome.[1,6]

An association with infertility has also been shown with the common SNP C677T of *MTHFR* gene in an Indian case-control study.[7] However, similar case-control studies for *MTHFR* C677T in European population have failed to record any correlation with male infertility.[8–10]

Thus the suggested association of the Y-chromosomal haplogroups, and/or polymorphisms in *MTHFR* gene with infertility indicates that certain genotypes/haplotypes affect fertility in region-specific manner.

The present study is addressed to the possible association of certain Y-haplotypes with infertility in the cases from eastern part of India. The Y-haplogroups chosen are those whose association with infertility has been demonstrated in Japanese and Danish populations.[1,6] However, the results failed to reveal any association of the haplogroups with infertility in the analysed population, though in the same cohort of cases and controls, C677T in the MTHFR gene had shown a strong association with infertility.

## Materials and Methods

### Subjects

In a case-control study, 165 patients classified as idiopathic azoospermic/oligospermic (157 azoospermic and 8 severe oligospermic with sperm count less than 1 million,mL) and 200 fertile control individuals of comparable age group (30, SD + 3), belonging to same geographical region were haplotyped for the binary alleles on the Y-chromosome. The cases were referred from the outpatient Endocrinology Clinic at the University Hospital and an *in vitro* fertilization (IVF) and a Urology Clinic, all in the city of Varanasi, India. Atleast three seminal fluid examinations, carried out after 3–4 days of sexual abstinence, were performed to ascertain there infertility status. Informed consent was obtained from all the subjects to carry out molecular analyses. Institutional ethical committee approval was obtained.

### Y Haplogroup analysis

**Figure 1:**
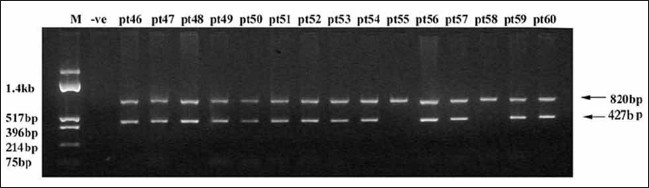
A representative illustration of haplotype analysis using 12f2 Indel marker. Haplotyping was done by PCR.[16] PCR assay generates a 427 bp product from chromosomes carrying the Taq I/10 kb allele but this product is absent from Taq I/8-kb allele chromosomes. An 800 bp amplicon from the SRY region, present in all the chromosomes, is amplified as a control. In the above agarose gel in pt #55 and pt #58 12f2 is deleted

**Figure 2:**
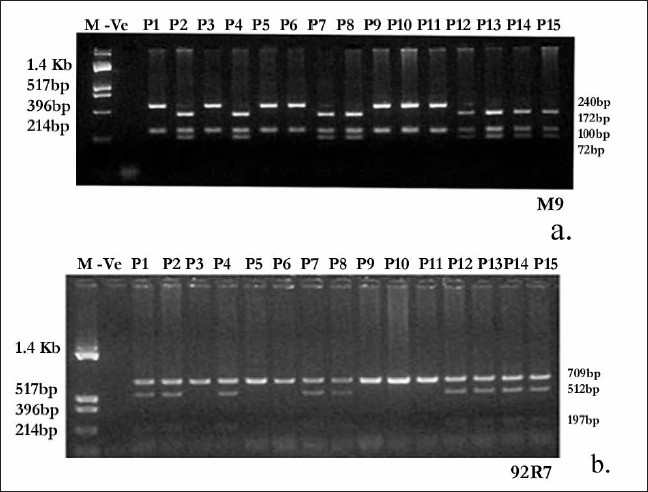
M9 (C→G) haplotype was detected by PCR-RFLP.[11] The 340 bp PCR product after digestion with *Hinf* I yields 172 bp, 100 bp and 68 bp product for the wild type C allele and 240 bp and 100 bp products for mutant G allele (a). 92R7 (C→T) was also haplotyped by PCR-RFLP.[12] The 709 bp PCR product was digested with *Hind* III. 709bp, 512 bp and 197bp fragments were obtained for the wild type C allele whereas 709 bp product for the mutant T allele as it was not cut by the enzyme (b)

**Figure 3:**
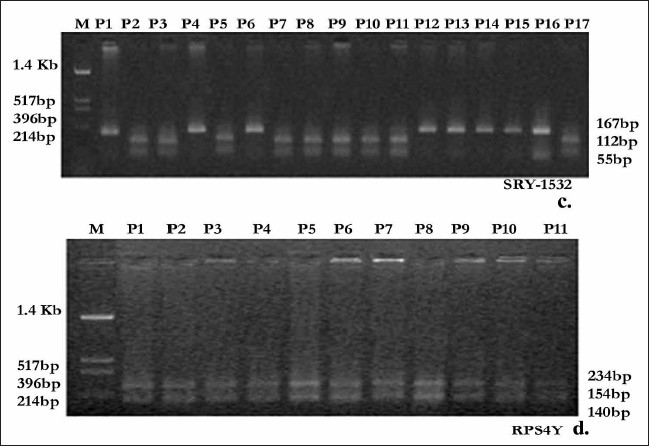
SRY 1532 (A→G→A) was haplotyped by PCR-RFLP method.[13,14] 167 bp PCR product after digestion with *Dra* III yields 112bp and 55 bp products for the wild type A allele and the mutant G allele was not cut by the enzyme (c). RPS4Y (C→T) was haplotyped by PCR-RFLP.[15] 528 bp PCR product was digested with Bsl I and after digestion for wild type C allele 234 bp, 154 bp and 140 bp products were obtained whereas for mutant T allele 388bp and 140 bp products

Genotyping of 4 SNPs and a 12f2 indel marker for all the subjects and controls were carried out by PCR-RFLP method [Figures 1-3]. The SNPs comprising different haplogroups were C→G (M9),[11] C→T (92R7),[12] A→G→A (SRY1532),[13,14] C→T (RPS4Y).[15] 12f2 deletion was genotyped.[16]

### Statistical analysis

Statistical analysis was carried out to assess the significance level of the distribution of haplogroups between the idiopathic azoospermic/oligospermic (n=165) and fertile male control population (n=200).

## Results

**Figure 4:**
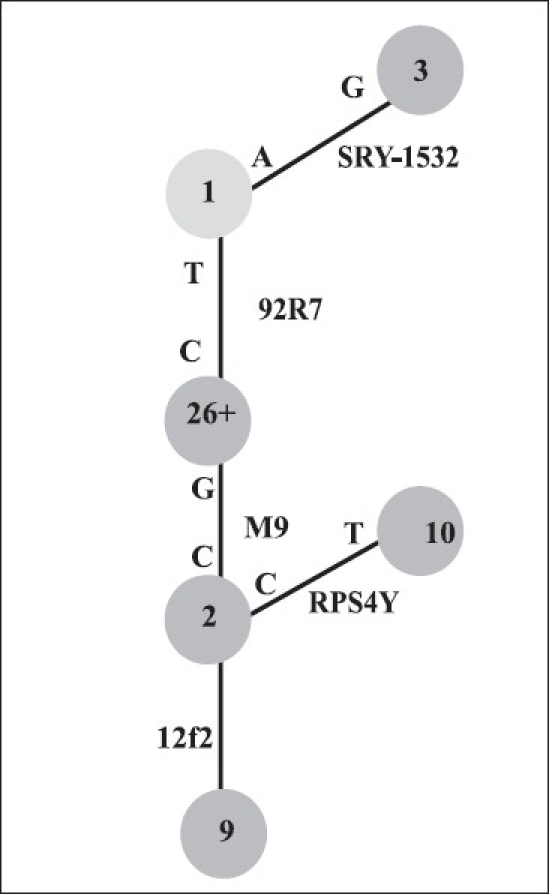
Phylogenetic tree of SNPs’ and Indel marker used in the study and the haplogroup they define

For Y-haplotyping, SNPs M9, 92R7, SRY-1532, RPS4Y and the Indel marker (12f2) were analysed. A parsimony network illustrating the relationships between the analysed haplogroups is shown in Figure 4. The haplogroup frequencies observed in azoo-/oligospermic infertile and fertile control men are summarized in Table 1. Out of the 6 haplogroup (Hg) markers used, only five were present in the studied population, Hg10, defined by RPS4Y711 C→T transition, being absent.[17] The association data as well as the frequency of alleles of different markers between the fertile and the azoo-/oligospermic subjects were subjected to χ^2^ statistical analysis. The test results revealed no statistically significant difference at the 5% level for both the groups. Similarly, the analysed haplogroups revealed no difference between the case and control samples [Table 1]. Also, there was no significant association in the distribution of any of the markers (M9, 92R7 and SRY-1532) with the case or the control populations.

**Table 1:** Haplogroup (hg) distribution in azoo-/oligospermic cases and control population

	hg1 (%)	hg2 (%)	hg3 (%)	hg26 (%)	hg9 (%)	Total
Azoo-/oligospermic	42(25.5)	42(25.5)	30 (18.2)	30 (18.2)	21 (12.7)	165
Control Population	38 (19)	52 (26)	42 (21)	49 (24.5)	19 (9.5)	200
Total	80	94	72	79	40	365

χ^2^ = 4.275, Df= 4, *P-value*>0.05

## Discussion

Compared with autosomal or mitochondrial DNA, the Y-chromosome shows a relatively higher level of geographic specificity. In the context of male infertility and Y-haplogroup, Hg 26+ shows a risk for infertility in a Danish population.[1] In another report, an association of Hg4 with low sperm count and infertility was found in a Japanese population.[6] However, a reassessment of Kuroki′s Hg 4 data along with other Y chromosome haplogroups (Hg20, 5, 2) from the same Japanese population did not find any association with infertility.[17] Similarly, a study on Italian subpopulations of infertile and control individuals initially indicated an association with specific haplogroups and idiopathic infertile males, but when samples were subdivided according to their origins the putative association between the two groups disappeared.[18] The present report too does not find any haplogroup's association with infertility. Hg26+, which showed association in the Danish population, had only a minor presence in both groups in the present study. The fact that so far only Hg26+ has shown a clear association suggests that there is need for exploring more haplogroups in more populations, and that a more rigorous analysis of multiple haplogroups may be necessary to derive meaningful conclusions.

In this paper we have found no association of infertility with the Y-chromosome haplogroups in an Indian population. Nevertheless, we maintain that such association studies need to be extended in their scope and in diverse populations to get a better perspective of the genotype-environment interaction in this multifactorial disorder.
